# Therapeutic effects of segmental resection and decompression combined with joint prosthesis on continuous knee osteoarthritis

**DOI:** 10.12669/pjms.306.5341

**Published:** 2014

**Authors:** Junlai Xue, Changhong Wang, Peng Liu, Xiangchun Xie, Shan Qi

**Affiliations:** 1 Junlai Xue, China-Japan Union Hospital, Changchun 130033, P. R. China; 2 Changhong Wang, China-Japan Union Hospital, Changchun 130033, P. R. China; 3 Peng Liu, China-Japan Union Hospital, Changchun 130033, P. R. China; 4 Xiangchun Xie, China-Japan Union Hospital, Changchun 130033, P. R. China; 5 Shan Qi, China-Japan Union Hospital, Changchun 130033, P. R. China

**Keywords:** Knee osteoarthritis, Joint replacement, Segmental resection and decompression, Joint prosthesis, Complication

## Abstract

***Objective:*** To observe the therapeutic effects of segmental resection and decompression combined with joint prosthesis on continuous knee osteoarthritis (OA)**.**

***Methods:*** A total of 130 patients with knee OA were selected and randomly divided into an observation group and a control group (n=65). The control group was treated by segmental resection in combination with joint prosthesis, and the observation group was treated by segmental resection and decompression combined with joint prosthesis. They were followed-up for three months.

***Results:*** All patients underwent successful surgeries during which no severe complications occurred. During the follow-up period, the overall effective rates of the observation group and the control group were 93.8% and 78.5% respectively, which were not statistically significantly different (p < 0.05). The observation group was significantly less prone to patellar instability, infection and deep vein thrombosis compared with the control group (P < 0.05). On the same day after surgery, the knee joint scores and functional scores of the two groups were similar, which evidently increased three months later, with significant intra-group and inter-group differences (p < 0.05).

***Conclusion: ***Combining segmental resection and decompression with joint prosthesis gave rise to satisfactory short-term prognosis by effectively improving the flexion and extension of injured knee and by decreasing complications, thus being worthy of promotion in clinical practice.

## INTRODUCTION

Knee joint is most prone to diseases due to complex structure and maximum burden in human body.^1^ Therefore, knee osteoarthritis (OA) has become the main disease endangering the elderly with population aging, thus being also known as senile arthritis.^2^ An epidemiological investigation showed that knee OA dominated in the reasons responsible for the labor deprivation of males older than 60, which was clinically manifested as joint pain, deformity and movement disorders that affected the quality of life.^3^ Trauma, gene, age and obesity are the risk factors of knee OA that is pathologically manifested as progressive destruction of involved joint cartilage, subchondral bone sclerosis and cartilage degeneration.^4^

Surgeries work well for knee OA treatment, of which segmental resection and decompression can relieve clinical symptoms and improve knee joint functions by drilling decompression of early degenerative joint.^5^ Joint replacement with prosthesis has been widely applied to treat knee OA, for which fixed-bearing prosthesis is always given first priority. The prosthesis can effectively improve the functions of knee joint.^6^ For the patients with continuous knee OA, however, even the internal fixation performed optimally is not enough without complete decompression due to the suppression of the spinal cord and nerve roots by protrusion of intervertebral disc and hyperostosis.

In this study, the therapeutic effects of segmental resection and decompression combined with joint prosthesis on knee OA were studied, aiming to provide clinical evidence for future reference.

## METHODS

***Study Subjects:*** A total of 130 patients with knee OA who were treated in our hospital from February 2011 to September 2013 were selected. This study has been approved by the institutional ethics committee of our hospital. ***Inclusion criteria:*** In accordance with the diagnosis criteria for knee OA; only one knee was involved; primary education level and above; without severe bone defects; 50-75 years old; without receiving internal or external treatment within two weeks before hospitalization; without history of knee joint surgery; without cerebrovascular, liver or kidney diseases; without severe hypertension or diabetes mellitus. The patients complicated with knee joint fracture, bone tumor, bone tuberculosis, rheumatoid, gout or purulent infections were excluded. Written consent was obtained from all patients. The patients comprised 72 males and 58 females aged (65.44 ± 3.12) years old (51~74 years old). The average disease course was (31.34 ± 8.33) months (1 month ~ 16 years). The flexion contractures ranged from 15° to 70°, with the average of (38.0 ± 10.23)°. The mean education duration was (16.34 ± 2.12) years. According to the clinical symptoms, there were 123 cases of knee joint pain, 67 cases of rest pain, 67 cases of atrophy of quadriceps femoris, and 56 cases of knee joint swelling. The mean body mass index (BMI) was (21.34 ± 1.89) kg/m^2^. They were randomly divided into an observation group and a control group, and their gender, age, disease course, flexion contracture, education duration and BMI were similar (p>0.05).

***Treatment methods: ***Prostheses were randomly selected without interfering factors. Replacement and decompression surgeries were conducted by the same panel of experienced surgeons according to requirements. LPS FLEX meniscal-bearing prosthesis (Zimmer, USA) was used.

Segmental resection and decompression were both performed for the observation group, whereas the control group was only subjected to segmental resection. After epidural anesthesia or general anesthesia, layers of tissues were incised in the middle of the anterior knee joint until the patella, in the center of which was drilled an approximately 8 mm vertical hole that extended below the cartilage after the flap was pulled open. After incision of the joint capsule, the patella was dislocated outwards while flexing the knee. Then the periosteum was dissected, and two rows of external bone cortex-perforating holes (three holes per row) were drilled 2 cm below the platform of medial condyle to perform depressive resection. Sharp dissections were carried out both inwards and outwards in the proximal patella, and a part of the infrapatellar fat pad was resected following regular protocols. A trial model was used before prosthesis implantation to simulate the responses after replacement. After the model was removed, the bone cross section was rinsed and dried.

Subsequently, the prostheses were fixed on the cross sections of the femur and the tibia. Afterwards, the stability and movement of the prostheses were re-checked. Homeostasis was conducted after the surgery, and drains were placed with anti-infection treatment. Then tissues were sutured layer-by-layer, and the incision was closed and subjected to sterilized dressing. Finally, the lower limb was bandaged with elastic bandages. Subsequently, the patients began to take exercises under instructions to recover the movements of knee joint, ankle joint and hip joint.^7^^,^^8^

***Observation indices:*** Surgical evaluation: The surgical time of knee joint replacement, as well as blood loss and blood transfusion case number during surgery were observed.

Outcome evaluation (three months after surgery): Cured: Disappearance of all symptoms such as pain and swelling, normal knee joint movement and negative tourniquet test; markedly effective: disappearance of most symptoms such as pain and swelling, unrestricted knee joint movement, and negative tourniquet test; effective: disappearance of almost all clinical symptoms, mildly restricted knee joint movement, and positive tourniquet test; ineffective: inability to meet the above requirements, and positive tourniquet test.

***Knee joint function evaluation:*** Knee joint score and functional score were evaluated three months after surgery based on the Knee Score System established in 1989 by American Association of Hip and Knee Surgeons.^9^

***Complications:*** The patients were followed-up for three months to observe complications such as patellar instability, infections and deep vein thrombosis.

***Statistical analysis: ***All data were analyzed by SPSS 13.5. The numerical data were expressed as mean ± standard deviation (x ± s). Comparisons were performed by t test and independent samples t-test. The categorical data were compared by Chi-square analysis. P<0.05 was considered statistically significant.

## RESULTS

***Surgical prognosis: ***All patients had the surgeries successfully, during which no severe complications occurred. The surgical time of knee joint replacement, as well as blood loss and blood transfusion case number during surgery were similar (P>0.05) (Table-I).

***Therapeutic effects:*** Three months after surgeries, the overall effectiveness of the observation group and the control group were 93.8% and 78.5% respectively, which were not statistically significantly different (P<0.05) (Table-II).

***Functional scores of knee joint: ***On the same day after surgery, the knee joint scores and functional scores of the two groups were similar, which evidently increased three months later, with significant intra-group and inter-group differences (P<0.05) (Table-III).

***Postoperative complications: ***During the follow-up period, the observation group was significantly less prone to patellar instability, infection and deep vein thrombosis compared with the control group (P<0.05) (Table-IV).

**Fig.1:**
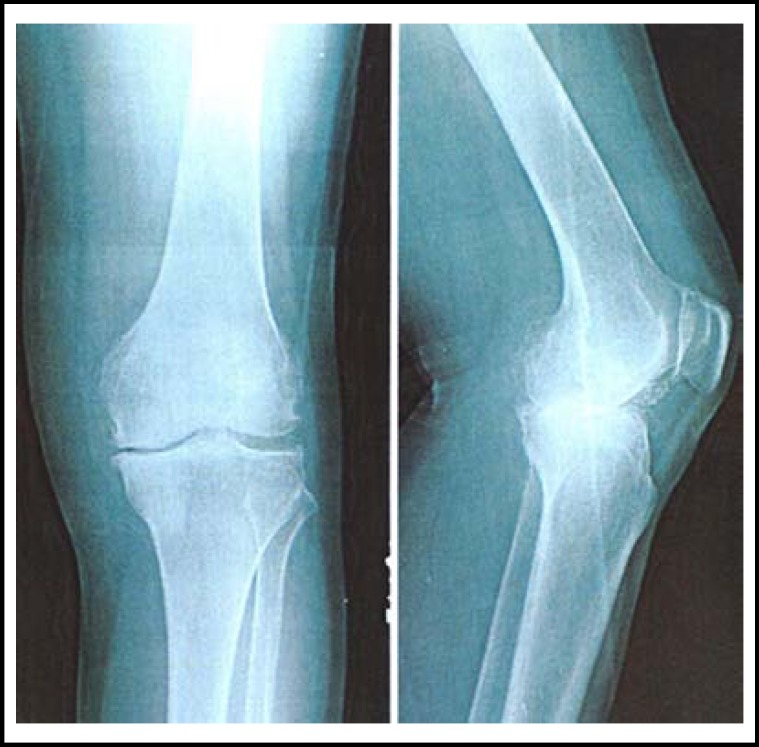
Lateral knee X-ray before surgery disclosed degenerative knee joint, osteophyte formation, degenerative patellofemoral joint, joint space narrowing and subchondral osteosclerosis

**Fig.2:**
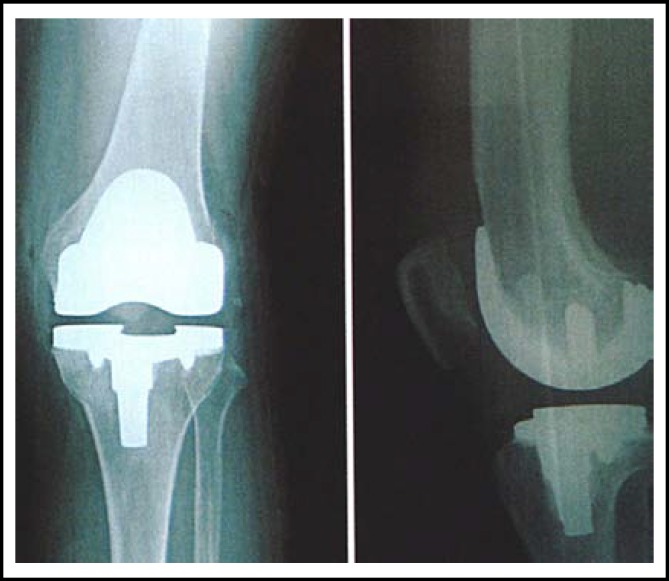
Lateral knee X-ray after surgery disclosed that there were no radiolucent regions at the interface between prosthesis and bone cement or that between bone cement and bone, and the prosthesis was firmly fixed in the ideal position.

***Case analysis: ***Ms. Wang, female, 65 years old, who had suffered from right knee OA for over six years. Both knees were painful, and the joint movement was apparently restricted. Three months after segmental resection and decompression combined with replacement of fixed-bearing prosthesis, the pain was relieved, accompanied by significantly improved joint movement (Fig.1 and Fig.2).

## DISCUSSION

It is well-known that knee joint burdens maximum loads in human body. Particularly, knee OA is a chronic arthropathy characterized as degeneration and destruction of joint cartilage as well as hyperostosis.^10^ The elderly are most prone to knee OA, and the prevalence rate of those older than 60 years old is approximately 50%. Moreover, such rate is increasing annually with population aging.^11^ Pathologically, knee OA begins in the joint cartilage, and thereafter subchondral bone, joint capsule and articular soft tissue are injured and structurally altered, thus stimulating synovial hyperplasia, inducing joint swelling, and aggravating pain and joint movement disorders.^12^ Upon knee OA, intraosseous pressure increases because venous drainage in the bone marrow of spongy cartilage is hindered, which raises the resistance to venous return and leads to nutritional blood flow disorders, thus damaging bone and cartilages.^13^

**Table-I:** Surgical prognosis

***Group***	***Case number (n)***	***Surgical time (min)***	***Blood loss (ml)***	***Blood transfusion case number (n)***
Observation group	65	124. 53±10. 34	354. 39±15. 08	4 (6. 2%)
Control group	65	125. 98±11. 73	356. 93±14. 08	5 (7. 7%)
χ^2^ or t		0. 187	0. 067	0. 056
P		>0. 05	>0. 05	>0. 05

**Table-II:** Therapeutic effects

**Group**	**Case No.**	**Cured**	**Markedly effective**	**Effective**	**Ineffective**	**Overall effective rate**
Observation group	65	45	16	4	0	93. 8%
Control group	65	31	20	10	4	78. 5%
χ^2^						9. 234
P						<0. 05

**Table-III:** Functional scores of knee joint (x ± s).

**Group**	**Case number (n)**	**Joint score**		**Functional score**	
		**0th day**	**Postoperative 3 months**	**0th day**	**Postoperative 3 months**
Observation group	65	16. 86±4. 29	48. 55±6. 32^	8. 57±3. 04	19. 45±4. 09^
Control group	65	16. 34±5. 12	32. 30±8. 31^	8. 56±2. 99	13. 23±5. 12^
t		0. 213	9. 344	0. 087	7. 334
P			<0. 05		<0. 05

^ Compared with the results on the same day after surgery, t = 15. 435, 15. 773, 6. 098 and 8. 113.

**Table-IV:** Postoperative complications (n).

**Group**	**Case No.**	**Patellar instability**	**Infection**	**Deep vein thrombosis**	**Total**
Observation group	65	1	1	0	2 (3. 1%)
Control group	65	3	3	3	9 (13. 8%)
χ^2^					3. 982
P					<0. 05

Currently, knee OA is treated by alleviating pain, delaying cartilage degeneration, improving joint function, and preventing or decreasing deformity. Traditionally, surgeries are performed to reduce intraosseous pressure and to mitigate pain and functional disorders.^14^ In contrast, segmental resection and decompression can relieve pain by sharply reducing the pressure through segmental drilling. Meanwhile, drilling slowly constructs collateral circulation by opening copious capillary beds, which alleviates venous stasis as a result. However, joint replacement is needed to maintain the therapeutic effects.^15^

As a novel surgical protocol, total knee replacement maintains correct prosthesis positions and soft tissue balancing and stability, targets osteotomy and prosthesis implantation accurately, and keeps equidistant joint space during flexion and extension. The ten-year survival rates of most prostheses have exceeded 95%, without causing pain or functional loss.^16^ In this study, there were no severe complications in the surgeries. The surgical time of knee joint replacement, as well as blood loss and blood transfusion case number during surgery were similar (p>0.05).

During decompression, the position of suppressed nerve root is located by imaging. Particularly, it is vital to entirely expose the anatomic landmark and to ensure surgical safety by separating outward the Longus colli muscle and by pulling it.^17^ Besides, decompression allows an appropriate matching between the femur and articular surface while maintaining high degree of flexion, as well as backward rolling of femur on the tibial plateau, thus improving the prognosis.^18^ In this study, the knee joint scores and functional scores of the two groups were similar on the same day after surgery, which remarkably increased three months later, with significant intra-group and inter-group differences (p<0.05). 

It is well-established that fixed-bearing prosthesis cannot simulate the kinematic characteristics of normal knee joint and therefore fails to circumvent the contradiction between low joint stress and free movement. In addition, relative motion between the femur and the tibia is bound to produce high shear force that loosens the interface between prosthesis and bone. However, lumbar spinal stenosis can be effectively treated by subtotal laminectomy and local decompression combined with intervertebral pedicle-screw internal fixation, and local decompression is reliable for interbody fusion surgeries.^19^ During the follow-up period, the observation group was significantly less subject to patellar instability, infection and deep vein thrombosis compared with the control group (p<0.05). Patellar instability, as a common complication in clinical practice, results from the unrestricted rotations of the tibia and the femur during flexion and extension. Hence, such patients are highly recommended to take functional exercise. Besides, as one of the most serious complications of joint replacement, postoperative infections may result in complete failure, to which particular attention should be paid. Furthermore, deep vein thrombosis, which results from reduced lower limb movement owing to severe knee joint deformity and pain, should be appropriately controlled and prevented.

In summary, combining segmental resection and decompression with joint prosthesis gave rise to satisfactory short-term prognosis by effectively improving the flexion and extension of injured knee and by reducing complications, thus being worthy of promotion in clinical practice.

## Authors’ Contributions:

**JX** **and** **SQ** designed the study and prepared the manuscript.

**CW, PL** **and** **XX **collected data and analyzed the results.
